# PDE4B Is a Homeostatic Regulator of Cyclic AMP in Dendritic Cells

**DOI:** 10.3389/fphar.2022.833832

**Published:** 2022-03-21

**Authors:** Amy M. Chinn, Cristina Salmerón, Jihyung Lee, Krishna Sriram, Eyal Raz, Paul A. Insel

**Affiliations:** ^1^ Department of Pharmacology, University of California, San Diego, La Jolla, CA, United States; ^2^ Department of Medicine, University of California, San Diego, La Jolla, CA, United States

**Keywords:** cyclic AMP (cAMP), compensation, phosphodiesterase (PDE), PDE4B, Th2 immunity

## Abstract

Chronic decreases in the second messenger cyclic AMP (cAMP) occur in numerous settings, but how cells compensate for such decreases is unknown. We have used a unique system—murine dendritic cells (DCs) with a DC-selective depletion of the heterotrimeric GTP binding protein Gα_s_—to address this issue. These mice spontaneously develop Th2-allergic asthma and their DCs have persistently lower cAMP levels. We found that phosphodiesterase 4B (PDE4B) is the primary phosphodiesterase expressed in DCs and that its expression is preferentially decreased in Gα_s_-depleted DCs. PDE4B expression is dynamic, falling and rising in a protein kinase A-dependent manner with decreased and increased cAMP concentrations, respectively. Treatment of DCs that drive enhanced Th2 immunity with a PDE4B inhibitor ameliorated DC-induced helper T cell response. We conclude that PDE4B is a homeostatic regulator of cellular cAMP concentrations in DCs and may be a target for treating Th2-allergic asthma and other settings with low cellular cAMP concentrations.

## Introduction

**FIGURE 1:**
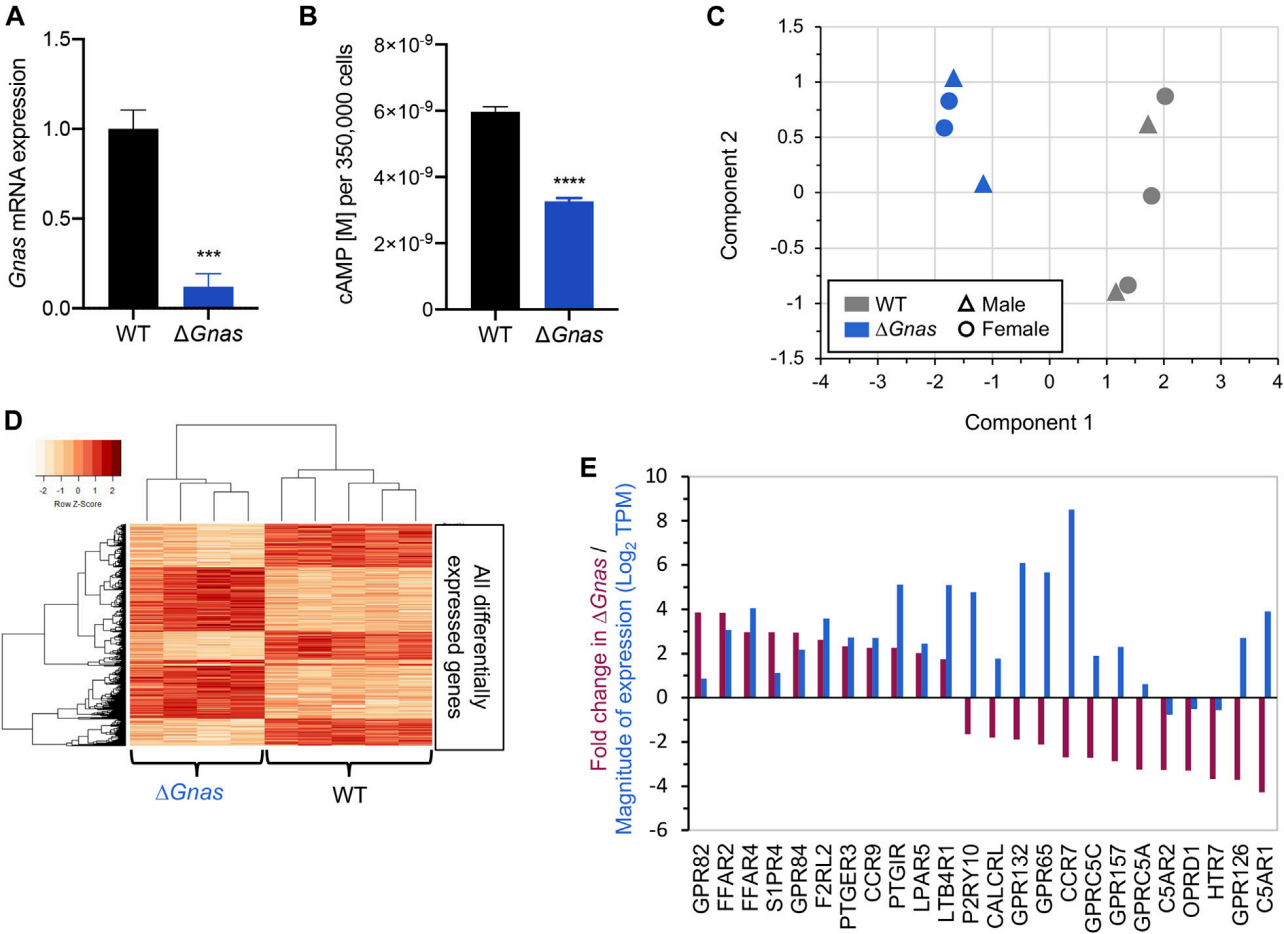
Δ*Gnas* DCs have decreased cAMP levels and associated changes in gene expression. **(A)** *Gnas* gene expression in wild-type (WT) and Δ*Gnas* DCs; *n* = 6, *p* < 0.001. **(B)** Basal cAMP levels in WT and Δ*Gnas* DCs; *n* = 5-6, *p* < 0.0001. **(C)** WT and Δ*Gnas* DC RNA-Seq samples as visualized in a Multidimensional Scaling (MDS) plot wherein a data set is analyzed to cluster the results into principal components (component 1 and component 2). These components explain the greatest degree of variance across the samples analyzed. The data show that WT and Δ*Gnas* DCs form two separate clusters based on gene expression. WT *n* = 5, Δ*Gnas n* = 4. **(D)** Heatmap of differentially expressed genes. Each column lists the gene expression from a single biological replicate. WT *n* = 5, Δ*Gnas n* = 4. **(E)** Fold-change (Purple) and level of expression (Blue) of differentially expressed GPCRs in Δ*Gnas* DCs compared to WT cells. WT *n* = 5, Δ*Gnas n* = 4, FDR< 0.05.

Cellular concentration of the second messenger 3′,5′-cyclic adenosine monophosphate (cyclic AMP, cAMP) can profoundly impact organismal physiology. Maintaining cellular cAMP homeostasis is thus essential (Raker et al., 2016). Decreased and increased cAMP levels in immune cells generally produce pro- and anti-inflammatory effects, respectively (Bariagaber and Whalen 2003; Bodor et al., 2012; Lee et al., 2015; Peters-Golden 2009; Rueda et al., 2016). Extracellular stimuli perturb intracellular concentrations of cAMP and trigger signaling cascades. Such perturbations are often transient: after removal of the stimulus, cAMP concentrations return to basal levels even though cellular microdomains may be sites of cAMP action (Bock et al., 2020; Tsvetanova et al., 2021; Zhang et al., 2020). By contrast, chronic dosing with drugs can persistently alter cAMP concentrations. Antagonists of G_s_-coupled G protein-coupled receptors (GPCRs) and agonists of G_i_-coupled GPCRs decrease cellular cAMP concentrations and are used to treat disease settings that include hypertension, congestive heart failure, and chronic pain (Law et al., 2000; Pierre et al., 2009; Rehsia and Dhalla 2010). Decreased cellular cAMP as a result of certain mutations in the gene *Gnas* result in clinical disorders, including pseudohypoparathyroidism, Albright hereditary osteodystrophy, and progressive osseus heteroplasia. Expression of certain cyclic nucleotide phosphodiesterases (PDEs) can increase in response to treatment with cAMP-elevating drugs; however, prior work has not defined how cells compensate for decreased cAMP levels (D’Sa et al., 2002; Lee et al., 2002; Liu et al., 2000). We hypothesized that previously unrecognized mechanisms may mediate compensation for a reduction in cAMP concentration and thereby contribute to cellular homeostasis. Here, we used a primary cell model to test this hypothesis: bone marrow-derived dendritic cells (DCs) from CD11c^Δ*Gnas*^ (Δ*Gnas*) mice that have a depletion of Gα_s_, an essential subunit of heterotrimeric (αβγ) G_s_ proteins that stimulate adenylyl cyclase to convert ATP to cAMP and activate cellular cAMP signaling (Figure 1A, Supplementary Figure S1A) (Lee et al., 2015; Lee et al., 2020). Our studies identify a novel role for PDE4B as a key contributor to cellular cAMP homeostasis and regulator of functional activity in DCs.

## Methods

### Animals

All animal procedures were approved by the University of California San Diego Institutional Animal Care and Use Committee (animal protocol S04173) and in accordance with the National Institutes of Health guide for the care and use of Laboratory animals. C57BL/6J mice and B6. Cg-Tg(TcraTcrb)425Cbn/J (OT-II) mice were purchased from the Jackson Laboratory. CD11c^Δ*Gnas*^ *(*Δ*Gnas)* mice were generated as described previously (Lee et al., 2015). DCs were isolated from both male and female mice for all experiments as RNA-Seq results did not indicate that samples clustered according to gender (Figure 1C).

### Bone Marrow-Derived Dendritic Cell (DC) Isolation

Mouse femurs and tibiae were cleaned of attached muscle and then briefly dipped in 70% ethanol. Bone marrow was collected by cutting the ends of the bones off with surgical scissors and flushing with 2% Fetal Bovine Serum (FBS, ThermoFisher Scientific, 16000044) in Phosphate Buffered Saline (PBS, ThermoFisher Scientific, 14040133). Cells were spun down at 1,220 rpm for 9 min at 4°C then passed through a 100 μm cell strainer (Corning, 431,752) and rinsed with RP10 media consisting of RPMI 1640 (ThermoFisher Scientific, 22400089) supplemented with 10% FBS (ThermoFisher Scientific, 16000044), 10% penicillin-streptomycin (ThermoFisher Scientific, 15140122), and 50 μM 2-Mercaptoethanol (Sigma Aldrich, M3148-100 ML). Cells were spun down at 1,220 rpm for 9 min at 4°C and then plated in Falcon Petri Dishes (Corning, 351,058) and cultured for 6 days at 5% CO_2_ in RP10 media with 10 ng/ml recombinant mouse Granulocyte-macrophage colony-stimulating factor (GM-CSF, ThermoFisher Scientific, 14833162). Cells were refed on day 4 of the culture with additional RP10 media and GM-CSF. On day 6, all the media and floating cells were removed and centrifuged at 1,220 rpm for 9 min at 4°C. CD11c^+^ DCs were then isolated using EasySep Mouse CD11c Positive Selection Kit II (StemCell Technologies, 18,780).

### Dendritic Cell-Induced T Cell Differentiation

Spleens from OT-II mice were removed and a single cell suspension of splenocytes was generated. Red blood cells were lysed using ACK Lysing Buffer (ThermoFisher Scientific, A1049201) before CD4^+^ T cells were isolated from this splenocyte population using EasySep Mouse CD4^+^ T cell Isolation Kit (StemCell Technologies, 19,852).

Isolated DCs were treated with 100 μg/ml ovalbumin (Sigma Aldrich, O1641) for 24 h and then co-cultured with 1 × 10^5^ isolated naïve OT-II CD4^+^ T cells in a 1:1 ratio for 3 days in RP10 media (see above formulation). CD4^+^ T cells were then removed from the culture and stimulated with 10 μg/ml plate-bound anti-CD3e antibody (clone 2c11) (BioXcell, BE0001-1) and 1 μg/ml anti-CD28 antibody (ThermoFisher Scientific, 16-0,289-81) for 24 h. Cytokine levels in the supernatant were assayed by ELISA (ThermoFisher Scientific, 88-7044-22 and 88-7371-22) per the manufacturer’s instructions.

To investigate the effects of PDE4 inhibitors on DC-induced T cell differentiation, WT or Δ*Gnas* DCs were isolated and then incubated with 100 μg/ml ovalbumin (Sigma Aldrich, O1641) and either 10 μM A33 (provided by Tetra Therapeutics, now commercially available at Tocris (Tocris, 6313)), 10 μM Ro 20-1724 (Sigma Aldrich, 557,502), or DMSO vehicle control for 24 h. DCs were then washed with RP10 media and co-cultured with OT-II CD4^+^ T cells. After 3 days, cytokine levels in the supernatant were assayed by ELISA.

### Cell Culture

DC2.4 cells were a kind gift from Kenneth Rock (cells first described in (Shen et al., 1997)). DC2.4 cells were cultured at 5% CO_2_ in RPMI 1640 (ThermoFisher Scientific, 21870076) supplemented with 10% FBS (ThermoFisher Scientific, 16000044), 2 mM L-glutamine (ThermoFisher Scientific, 25030081), 10% penicillin-streptomycin (ThermoFisher Scientific, 15140122), 1X NEAA (ThermoFisher Scientific, 11140050), 10 mM HEPES (ThermoFisher Scientific, 15630080), and 55 μM 2-Mercaptoethanol (Sigma Aldrich, M3148-100 ML).

WT and Kin^-^ S49 cells (Insel et al., 1975) were cultured in 10% CO_2_ in Dulbecco’s Modified Eagle Medium (DMEM) with 4.5 g/L glucose (ThermoFisher Scientific, 11960044) supplemented with 2 mM L-glutamine (ThermoFisher Scientific, 25030081), 10% heat-inactivated horse serum (Sigma Aldrich, H1138), 1 mM sodium pyruvate (ThermoFisher Scientific, 11360070), and 10 mM HEPES (ThermoFisher Scientific, 15630080). Cells were continuously maintained in logarithmic growth.

### Real-Time Quantitative PCR (qPCR)

DCs were isolated as stated above. For DCs that were treated with drugs, the cells were plated in a 24-well plate and cultured for the specified length of time at 5% CO_2_ in RPMI 1640 (ThermoFisher Scientific, 22400089) supplemented with 10% FBS (ThermoFisher Scientific, 16000044), 10% penicillin-streptomycin (ThermoFisher Scientific, 15140122), and 50 μM 2-Mercaptoethanol (Sigma Aldrich, M3148-100 ML). In different experiments, DCs were treated with Prostaglandin E_2_ (PGE_2_, Sigma Aldrich, P0409), CPT (8-(4-Chlorophenylthio)adenosine 3′,5′-cyclic monophosphate sodium salt; Sigma Aldrich, C3912), 6 MB (N6-Monobutyryladenosine 3′:5′-cyclic monophosphate sodium salt; Sigma Aldrich, M1380), 8 ME (8-(4-Chlorophenylthio)-2′-O-methyladenosine-3′, 5′-cyclic monophosphate, sodium salt; Axxora BLG-C041-05), MDL-12,330 A (Sigma Aldrich, M182), or vehicle control. After treatment, cells were washed 2x with DPBS before being lysed with RLT Buffer from the RNeasy Mini Kit (Qiagen, 74,104).

All RNA was isolated using RNeasy Mini Kit (Qiagen, 74,104) with on-column DNase digestion using RNase-Free DNase (Qiagen, 79,254). After isolation, 1 μg of RNA was converted into cDNA using iScript cDNA Synthesis Kit (Bio-Rad, 1708891). qPCR samples were run in triplicates on a Bio-Rad CFX Connect Real-Time PCR Detection System using PerfeCTa SYBR Green SuperMix (VWR, 101414-146) with 0.5 μM primers and 8 ng cDNA in a 20 μl reaction volume. dCt values were calculated by subtracting the Ct value for the housekeeping gene 18S from the Ct value of the gene being evaluated. A dCt <25 was used as the threshold of detection. Fold-change between two genes was calculated by formula 2^-^(^dCT^Gene 1 ^– dCt^Gene 2). Unless otherwise stated, data were normalized to WT or vehicle control.

Primers were designed with Primer-BLAST using thermodynamic oligo alignment and analyzed with Integrated DNA Technologies (IDT) OligoAnalyzer Tool to minimize self- and heterodimer tendencies for each primer set. Primer sequences are shown in Supplementary Table S3.

### RNA-Sequencing Analysis and Data Mining

Libraries for RNA-Sequencing (RNA-Seq) were prepared at the University of California San Diego Institute for Genomic Medicine Core Facility, using the Illumina Truseq stranded mRNA kit (Illumina, 20022371) per manufacturer’s protocols. Libraries were sequenced using a NovaSeq 6000 sequencing system (Illumina) as per manufacturer’s protocols, with 150 base pair, paired-end reads, at the University of California, San Francisco Center for Advanced Technology core facility.

Following sequencing, FASTQ files were analyzed as follows. First, files were inspected for sequencing quality using FASTQC (www.bioinformatics.babraham.ac.uk/projects/fastqc/). All files cleared standard quality assessment, with no quality trimming or other steps needed, apart from removal of adapters. Adapters were trimmed using BBDuk, using standard commands (jgi.doe.gov/data-and-tools/bbtools/bb-tools-user-guide/bbduk-guide/) and removing sequences corresponding to known Illumina adapters, yielding cleaned-up FASTQ files, which were rechecked via FASTQC to verify adapter removal.

From these files, we quantified gene expression via Kallisto (v0.43.1) with bias detection on (Bray et al., 2016), using the Ensembl v79 reference transcriptome for mice. Transcript expression from Kallisto was converted to gene-level expression (in transcripts per million [TPM] and estimated counts) via the Tximport package (Soneson et al., 2015) in R, using standard commands in the Tximport vignette. Estimated counts were used as input to the edgeR package (Robinson et al., 2010) in R, counts were normalized using the “TMM” method (trimmed mean of M-values), followed by assessment of differential expression (DE), via the inbuilt “exact test” method in edgeR. Low expressed genes (i.e., those not expressed >1 count per million in at least three samples) were filtered out prior to DE analysis. Genes with false discovery rate (FDR) < 0.05 were considered to have statistically significant DE. RNA-Seq data of WT and Δ*Gnas* DCs were deposited in NCBI’s Gene Expression Omnibus under accession number GSE158783.

Single-cell RNA-Seq (scRNA-Seq) analysis of WT CD11c^+^/ CD45.1^+^/CD45.2^+^ DCs from mouse lungs were obtained from GEO, accession number GSE149617 (PMID 32392463). Processed data from 10x scRNA-Seq of these cells were available from GEO, with filtered UMIs (unique molecular identifiers) stored as h5 files. These h5 files were analyzed in R, via Seurat (PMID: 31178118). In brief, files were converted to Seurat objects in R, followed by normalization using relative counts, with a scale factor = 10,000. These normalized counts were used as input for Supplementary Figure S2A which shows expression for PDEs. In addition, the data were rescaled using the SCtransform tool within Seurat, followed by dimension reduction via UMAP (Uniform Manifold Approximation and Projection) using 30 dimensions. The UMAP reduced data were then plotted using the FeaturePlot tool in Seurat to show the spatial variation of PDE expression in the DC population analyzed in Supplementary Figure S2B.

Data from RNA-Seq analysis of human epidermal dendritic cells were obtained from GEO, accession number GSE130804 (Bertram et al., 2019). Processed data in gene-level counts were available from GEO; these data were analyzed via edgeR (Robinson et al., 2010), allowing for expression to be normalized via the TMM method (as above) and expressed in counts per million (CPM). Expression for PDEs was extracted from these normalized data in CPM and plotted in Figure S2C. This dataset had *n* = 4 donors with four replicates of CD11c^+^ DC samples sequenced from each donor, yielding n = 16 samples.

### Cyclic AMP Assay

Isolated CD11c^+^ DCs were seeded at 350,000 cells/well in a white bottom, white walled 96-well plate (Millipore Sigma, M0187) and cultured overnight in RP10 media at 37°C, 5% CO_2_. To assay basal cAMP levels, media was removed and the assay performed on the cells attached to the bottom of the wells so as to only measure intracellular cAMP. Intracellular cAMP levels were then assayed by the HitHunter cAMP Assay for Biologics (DiscoverX, 90-0075LM25) per the manufacturer’s instructions.

### Western Blot

Isolated CD11c^+^ DCs were washed twice with PBS and re-suspended in RIPA buffer (Cell Signaling Technology, 9806S) with 1X protease inhibitor cocktail (Sigma Aldrich, P8340-1 ML) and 1X phosphatase inhibitor cocktail (PhosSTOP, Sigma Aldrich, 4906845001). Samples were sonicated 3X and then centrifuged at max speed for 15 min at 4°C. Supernatants were removed and stored at −70°C. Protein was quantified by Pierce BCA Protein Assay Kit (ThermoFisher Scientific 23,225). Samples were mixed with 4x Laemmli Sample Buffer (Bio-Rad, 1610747) with 2-mercaptoethanol and boiled at 95°C for 10 min. 20 μg of protein was loaded on a 12% Criterion TGX Precast Protein Gel (Bio-Rad, 5671043) and run using Bio-Rad Criterion Vertical Electrophoresis Cell on 85 V for 10 min, and then 15 W, max amp, 200 V for 45 min. Precision Plus Protein Kaleidoscope Prestained Protein Standards (Bio-Rad, 1610375) was used for the ladder. Membranes were activated in methanol and blots were transferred with Bio-Rad Criterion Blotter (100 V for 45 min). Membranes were blocked in 5% milk/1X TBST (Cell Signaling Technology, 9997S) at room temperature and incubated with primary rabbit anti-GNAS antibody (Abcam, ab83735; 1:500 dilution) in 2.5% milk/TBST at 4°C overnight on a shaker. Blots were then washed with 1X TBST, incubated with secondary anti-Rabbit-HRP (Cell Signaling, 70,745; 1:5000 dilution) in 2.5% milk/TBST for 1 h at room temperature on a shaker, washed with TBST, and then visualized using 1 ml of ECL Ultra chemiluminescent reagent (Lumigen, TMA-6) on a Fluorchem HD2 imaging system, using Alphaview 3.4.0.0 software.

Blots were then stripped with Restore Western Blot Stripping Buffer (ThermoFisher Scientific, 21,059), washed, blocked with 5% milk/1X TBST, and re-incubated with goat anti-β-tubulin antibody (Abcam, ab21057; 1 μg/ml) in 2.5% milk/TBST at 4°C overnight on a shaker. Blots were then washed with 1X TBST, incubated with secondary anti-Goat-HRP antibody (Abcam, ab6013; 1:5000 dilution) for 1 h at room temperature on a shaker, washed with TBST, and then visualized. Afterwards, they were stripped (ThermoFisher Scientific, 21,059) per manufacturer’s instructions, washed, blocked with 5% milk/1X TBST, and re-incubated with mouse anti-PDE4B antibody (LSBio, LS-B11018; 1:500 dilution) in 2.5% milk/TBST at 4°C overnight on a shaker. Blots were then washed with 1X TBST, incubated with secondary anti-mouse-HRP antibody (Cell Signaling, 70,765; 1:3,000 dilution) for 1 h at room temp on a shaker, washed with TBST, and visualized. ImageJ was used to analyze densitometry.

### Phosphodiesterase (PDE) Activity Assay

PDE activity was measured using Cyclic Nucleotide phosphodiesterase Assay Kit (Enzo Life Sciences, BML-AK800-0,001). Isolated CD11c^+^ DCs were resuspended in the PDE Assay Buffer and 1X protease inhibitor cocktail (Sigma Aldrich, P8340-1 ML) and sonicated. Samples were then centrifuged at 10,000 rpm for 10 min at 4°C. Lysates were removed and desalted per the manufacturer’s instructions before being stored at −70°C. Protein was quantified by Pierce BCA Protein Assay Kit (ThermoFisher Scientific, 23,225). PDE activity in 2.5 μg protein was assayed per the manufacturer’s instructions.

### Statistical Analysis

Data were analyzed with Prism 8.0 software (GraphPad Software, La Jolla, CA) and are presented as mean ± SEM. Statistical analysis was done using two-way ANOVA with Sidak’s multiple comparisons test to correct for multiple comparisons. Unpaired t tests were used to determine statistical significance when comparing two groups. A value of *p* < 0.05 was considered statistically significant.

## Results

Δ*Gnas* mice, generated by crossing *Gnas* ^fl/fl^ mice with CD11c-Cre mice, have reduced basal cAMP levels (Figure 1B) and cAMP accumulation in response to stimulation with G_s_-coupled GPCR agonists and forskolin (Lee et al., 2015). The small amount of *Gnas* present in Δ*Gnas* DCs (Figure 1A) is due to the leakiness of CD11c-Cre (Lee et al., 2015). Δ*Gnas* DCs thus have persistent, low cAMP concentrations and provide a primary cell model to investigate compensation for chronically decreased cAMP levels. RNA-Seq of WT and Δ*Gnas* DCs revealed that WT and Δ*Gnas* DCs cluster separately from one another (Figure 1C). Differentially expressed genes of WT and Δ*Gnas* DCs form distinct groups, and groups of co-regulated genes form a consistent pattern between the two genotypes (Figure 1D).

To assess for compensatory changes, we used RNA-Seq and qPCR to evaluate upstream and downstream components of the cAMP signaling pathway. We found that WT and Δ*Gnas* DCs express a similar number of GPCRs (Supplementary Tables S1,S2), with G_i_-linked GPCRs being the largest group of GPCRs (Supplementary Figures S1B,C). Twenty GPCRs had >2-fold increased or decreased expression in Δ*Gnas* DCs (FDR< 0.05) (Figure 1E). Seven of these decreased GPCRs couple to G_i_ (*S1PR4*, *GPR84*, *PTGER3*, *CCR9*, *CCR7*, *OPRD1*, *C5AR1*) and are predicted to inhibit adenylyl cyclase activity and might contribute to compensation for decreased cAMP levels by blunting further decrease of cAMP. Expression of Gα proteins (other than Gα_s_) and adenylyl cyclase isoforms were not significantly different in Δ*Gnas* DCs (Supplementary Figures S1D–H).

**FIGURE 2:**
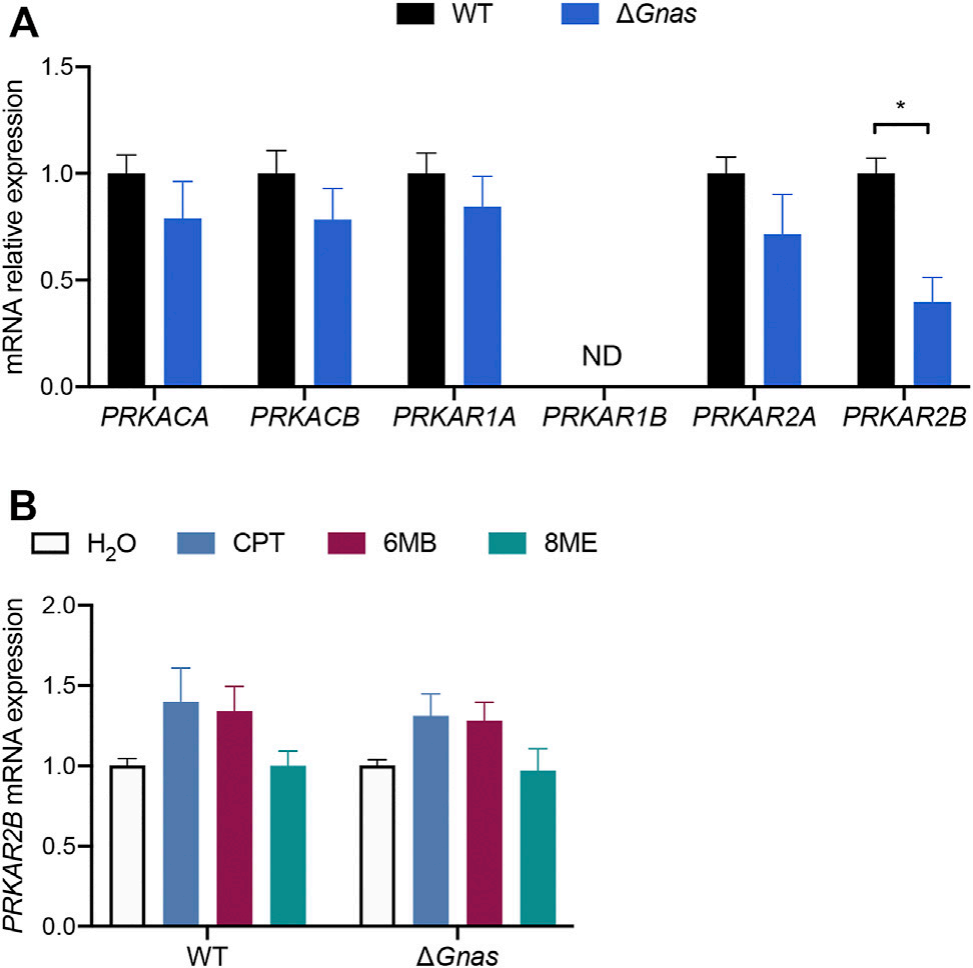
Δ*Gnas* DCs have decreased expression of *PRKAR2B,* whose expression is insensitive to increases in cAMP levels. **(A)** Protein kinase A (PKA) subunit expression in WT and Δ*Gnas* DCs; n = 5, *p* < 0.05. **(B)** *PRKAR2B* expression in WT and Δ*Gnas* DCs after treatment with the cAMP analogs CPT (non-selective), 6MB (PKA-selective), and 8ME (Epac-selective), (each 50 μM, 24 h); *n* = 3, data are not statistically significant.

cAMP primarily mediates its effects via protein kinase A (PKA) and exchange protein directly activated by cAMP (Epac). The two Epac genes, *EPAC1* and *EPAC2,* were not detectable, implicating PKA as the mediator of cAMP signaling in murine DCs. Δ*Gnas* DCs had >55% reduction in *PRKAR2B* mRNA which encodes the PKA regulatory subunit RIIβ, suggesting possible feedback between decreased cAMP concentration and its expression (Figure 2A, Supplementary Figure S1I). However, elevation in cAMP levels did not increase PKA RIIβ expression in Δ*Gnas* DCs treated for 24 h with CPT (a non-selective cAMP analog), 6MB (a PKA-selective cAMP analog), and 8ME (an Epac-selective cAMP analog) (Figure 2B). A-kinase anchoring proteins (AKAPs) are scaffolds that tether protein complexes with PKA in subcellular regions (Esseltine and Scott 2013). Expression of the 14 murine AKAPs was unaltered in Δ*Gnas* DCs (Supplementary Figures S1J,K).

**FIGURE 3:**
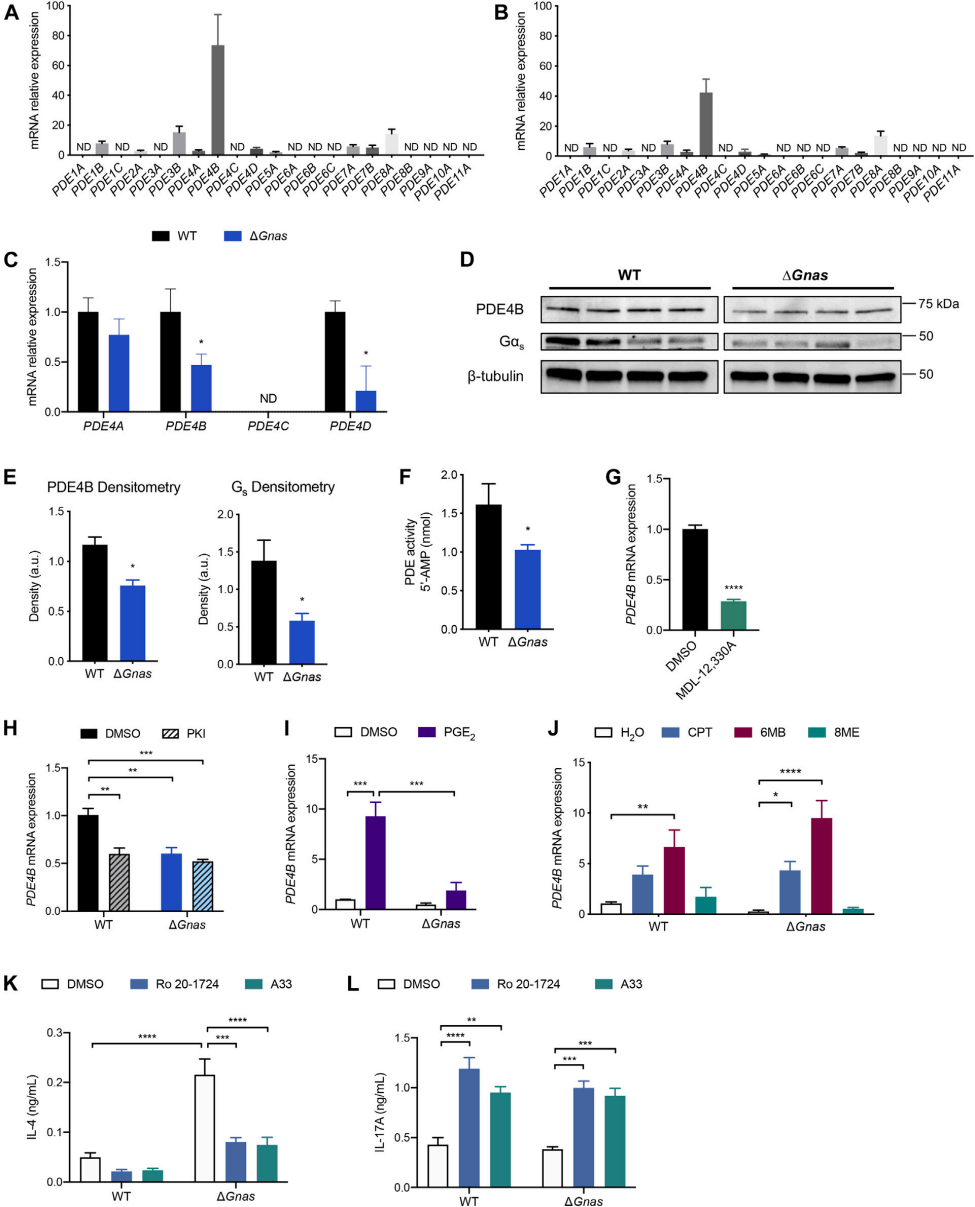
PDE4B expression regulates cAMP levels and PDE4B inhibition decreases Th2 differentiation and increases Th17 differentiation in DCs. **(A**,**B)** Expression profile of PDE mRNA expression in **(A)** WT DCs; *n* = 7 and **(B)** Δ*Gnas* DCs; *n* = 6. ND = not detected; data normalized to lowest-expressing detectable gene. **(C)** PDE4 isoform fold change in Δ*Gnas* DCs compared to WT cells; *n* = 5–6. **(D)** Western blots and **(E)** densitometry of Gα_s_ and PDE4B protein expression in WT and Δ*Gnas* DCs; *n* = 4. **(F)** PDE activity in WT and Δ*Gnas* DCs; *n* = 5–6. **(G)** *PDE4B* expression after treatment of WT DCs with the adenylyl cyclase inhibitor MDL-12,330A (10μM, 16 h); *n* = 3. **(H)** *PDE4B* expression in WT and Δ*Gnas* DCs treated with a PKA inhibitor (PKI, 10 μM) for 24 h; *n* = 4. Data normalized to WT DMSO control. **(I**,**J)** Fold change in *PDE4B* expression after treatment of WT and Δ*Gnas* DCs with **(I)** PGE_2_ (10μM, 24 h); n = 2-4, and **(J)** cAMP analogs CPT (non-selective, 50 μM, 24 h), 6MB (PKA-selective, 50 μM), and 8ME (Epac-selective, 50 μM) for 24 h; *n* = 3–5. Data normalized to WT vehicle control. **(K,L)** WT and Δ*Gnas* DCs were treated with DMSO (vehicle control), Ro 20-1724 (pan-PDE4 inhibitor, 10 μM), or A33 (PDE4B-selective inhibitor, 10 μM) for 24 h before culturing with CD4^+^ OT-II T cells. **(K)** IL-4 secretion and **(L)** IL-17 A secretion from co-cultured T cells was quantified; *n* = 4. *p < 0.05, **p < 0.01, ***p < 0.001, ****p < 0.0001.

Cyclic AMP is removed from cells via efflux by the multidrug resistance-associated protein 4 (MRP4/ABCC4) transporter and via hydrolysis by PDEs. *MRP4* expression was not changed in Δ*Gnas* DCs (Supplementary Figures S1L,M). Assessment of the 21 murine PDEs revealed that PDE4B is the predominant isoform in WT and Δ*Gnas* DCs and that PDE4B is expressed many-fold higher than other PDE isoforms (Figures 3A,B). Single cell RNA-Seq data of mouse lung DCs (Bosteels et al., 2020) (Supplementary Figures S2A,B) and bulk RNA-Seq data of human epidermal DCs (Bertram et al., 2019) (Supplementary Figure S2C) confirm that PDE4B is the highest expressed PDE isoform in both murine tissue-resident and human DCs. We found that Δ*Gnas* DCs have decreased expression of 2 PDEs: *PDE4B* is reduced >50% and *PDE4D* is reduced 60% compared to WT cells (Figure 3C). Since *PDE4B* is predominant in DCs and 20-fold higher expressed than *PDE4D*, our subsequent studies focused on PDE4B. Δ*Gnas* DCs also had reduced PDE4B protein expression and decreased overall PDE activity, consistent with the prominent expression of *PDE4B* mRNA in DCs (Figures 3D–F). RNA-Seq data revealed that murine DCs express 4 PDE4B transcripts; the 2 longest transcripts account for >85% of *PDE4B* expression in both WT and Δ*Gnas* DCs with no differences between genotypes in the prevalence of transcript variants (Supplementary Figures S2D-F).

Decreased cAMP in WT DCs in response to treatment with the adenylyl cyclase inhibitor MDL-12,330A lowered *PDE4B* mRNA expression, demonstrating that reduced *PDE4B* expression in Δ*Gnas* DCs stems from the decrease in cAMP and not from depletion of Gα_s_ (Figure 3G, Supplementary Figure S3A). Treatment of WT DCs for 24 h with PKI, an endogenous PKA inhibitor, reduced *PDE4B* mRNA, implying that PKA mediates the decrease in PDE4B expression (Figure 3H). To test if reduced PKA activation decreases *PDE4B* levels in other cell types, we assessed its expression in WT and Kin^-^ S49 T lymphoma cells. Kin^-^ S49 cells, which lack PKA activity but have functional Gα_s_ (Orellana and McKnight 1990), have an 80% decrease in *PDE4B* expression compared to WT S49 cells, consistent with the findings in Δ*Gnas* DCs (Supplementary Figure S3B).

To determine if PDE4B expression is bidirectionally regulated by cAMP, we treated WT and Δ*Gnas* DCs with PGE_2_ for 24 h. PGE_2_ treatment increased *PDE4B* expression in WT DCs, a response that was blunted in Δ*Gnas* DCs (Figure 3I). To investigate which cAMP effector mediated this increase in expression, we treated WT and Δ*Gnas* DCs for 24 h with the cAMP analogs CPT and 6MB, but not 8ME, increased *PDE4B* expression in WT and Δ*Gnas* DCs, thus implicating PKA as the mediator of the increase in *PDE4B* expression (Figure 3J). We obtained similar results using DC2.4 cells (a DC cell line): increased cAMP via PKA increased *PDE4B* levels (Supplementary Figures S3C,D). Thus, expression of *PDE4B* is dynamic and regulated by cAMP/PKA, decreasing and increasing in cells with lower and higher cAMP concentrations, respectively.

We next assessed whether PDE4B influences DC function, in particular the ability of DCs to regulate Th2 immunity (Lee et al., 2015). Increased cAMP levels have an anti-inflammatory effect on DCs, so antagonism of PDE4B could potentially reduce DC-induced Th2 inflammation (Galgani et al., 2004; Jin et al., 2010; Lee et al., 2015; Rueda et al., 2016; Chinn and Insel 2020). Δ*Gnas* DCs have reduced PDE4B expression compared to WT cells, but PDE4B is still the predominant PDE in the Gα_s_ *-*depleted cells (Figure 3B, Figures 3D,E).

Allergic asthma is largely driven by Th2-mediated inflammation (Fajt and Wenzel 2015; Tran et al., 2016). Δ*Gnas* mice develop allergic asthma in response to ovalbumin (OVA) immunization, as shown by increased IgE serum levels, airway hyperresponsiveness, and airway inflammation (Lee et al., 2015). These mice also spontaneously develop asthma at age 6 months without OVA immunization, mimicking the spontaneous development of asthma in humans. Allergic asthma in Δ*Gnas* mice is driven by preferential differentiation of Th2 cells by Δ*Gnas* DCs. By contrast, WT DCs do not induce naïve CD4^+^ T cells to differentiate into Th2 cells *ex vivo* (Lee et al., 2015).

We treated WT and Δ*Gnas* DCs with ovalbumin plus either vehicle control, the pan-PDE4 inhibitor Ro 20-1724, or the PDE4B-selective inhibitor A33 (Naganuma et al., 2009). DCs were then co-cultured with naïve CD4^+^ T cells from OT-II mice to determine if PDE4 inhibition in DCs affects helper T cell differentiation. Treatment of Δ*Gnas* DCs with either Ro 20-1724 or A33 equally decreased IL-4 secretion by co-cultured CD4^+^ T cells, implying that PDE4B likely accounts for the majority of the PDE4 activity in Δ*Gnas* DCs and that PDE4/PDE4B inhibition in DCs reduces Th2 differentiation (Figure 3K). Decreased DC-induced Th2 differentiation can abrogate allergic lung inflammation *in vivo* (Lee et al., 2015). Thus, targeting PDE4B may be a novel means to reduce DC-mediated Th2 inflammation–even in DCs with decreased expression from compensation for lower cAMP concentrations. Ro 20-1724 and A33 treatment of WT and Δ*Gnas* DCs also increased T cell-secreted IL-17 A which is indicative of increased Th17 differentiation, consistent with data showing that increased cAMP levels in DCs promote Th17 differentiation (Figure 3L) (Datta et al., 2010; Lee et al., 2020).

## Discussion

Altered concentrations of cAMP can regulate many cellular functions and phenotypes; maintaining cAMP homeostasis is thus a critical feature of cell and tissue physiology (Raker et al., 2016; Chinn and Insel 2020). Moreover, altering cAMP levels is used to treat many diseases, including pulmonary, cardiovascular, renal, gastrointestinal, dermatologic, and neuropsychiatric disorders (Law et al., 2000; Pierre et al., 2009; Rehsia and Dhalla 2010). Prior work has demonstrated that cells can compensate (decrease toward basal levels) drug-induced increases in cAMP by increasing RNA expression of PDE isoforms (D’Sa et al., 2002; Le Jeune et al., 2002; Catherin jin and Conti 2002; Liu et al., 2000; Torphy et al., 1995). The current data identify a mechanism by which cells can compensate for decreased cAMP in a primary cell system, in particular in DCs, and have implications for cellular homeostasis with respect to settings with altered cAMP levels.

The current data are the first of which we are aware that have defined PDE expression and functional responses in DCs. A recent review that discussed roles for PDE4 in multiple immune cell types did not include discussion of DCs (Zuo et al., 2019). The prominent expression of PDE4B in DCs is consistent with its detection in other immune and inflammatory cells (Jiang et al., 1998; Catherin jin and Conti 2002; Maurice et al., 2014).

How does cAMP regulate PDE4B expression? *PDE4B* has a CRE site in its promoter region, suggesting its expression is mediated by a CREB family member (D’Sa et al., 2002; Wang et al., 2003). Our findings support the idea that PDE4B is responsible for 1) reduced PDE activity in Δ*Gnas* DCs, and 2) compensation to increase cAMP levels in a setting of persistent decrease in cAMP concentration. The decrease in PDE4B expression does not restore “normal” cAMP levels, since cAMP concentration in Δ*Gnas* DCs is less than that of WT DCs, but the decrease in PDE4B may partially blunt physiological effects resulting from decreased cAMP levels. PDE4B thus acts as a homeostatic regulator of cellular cAMP concentrations, rising with increased and falling with decreased cAMP levels. Other cell types may use additional compensatory mechanisms, e.g., elevated cAMP can increase *MRP4* mRNA in HeLa and vascular smooth muscle cells via Epac-mediated actions (Bröderdorf et al., 2014).

Many marketed drugs alter cAMP levels. Our findings suggest a targeted strategy directed at components that maintain cAMP homeostasis. PDE4B appears to be a key regulator of both increased and decreased cAMP levels in DCs; other components might be targeted in other cell types. PDE4 is implicated in multiple disease settings, with 3 FDA-approved PDE4 inhibitors marketed for the treatment of atopic dermatitis, chronic obstructive pulmonary disorder, psoriasis, and psoriatic arthritis; several PDE4B-selective inhibitors are in development (Li, Zuo, and Tang 2018; Tibbo and Baillie 2020). These and other findings support the evidence that PDE4B and other PDEs are key contributors to cellular homeostasis, including in cellular microdomains/nanodomains (Bock et al., 2020; Zhang et al., 2020). PDEs, including PDE4B in at least certain cell types, thus appear to be fundamental for modulating cellular function and ultimately organismal health. In this regard, the current study identifies a potential role of PDE4B inhibition as a therapeutic approach to increase cAMP in DCs as a means to blunt Th2-mediated responses.

## Data Availability

The datasets presented in this study can be found in online repositories. The names of the repository/repositories and accession number(s) can be found below: https://www.ncbi.nlm.nih.gov/, GSE158783.
