# Supplementary material for: PDE4B Is a Homeostatic Regulator of Cyclic AMP in Dendritic Cells
Source: Front Pharmacol. 2022 Mar 21;13:833832. doi: 10.3389/fphar.2022.833832 (PMC8977838; doi:10.3389/fphar.2022.833832)
Supplement: Supplementary file 1 [file DataSheet1.pdf]

Supplementary Material

1 Supplementary Figures

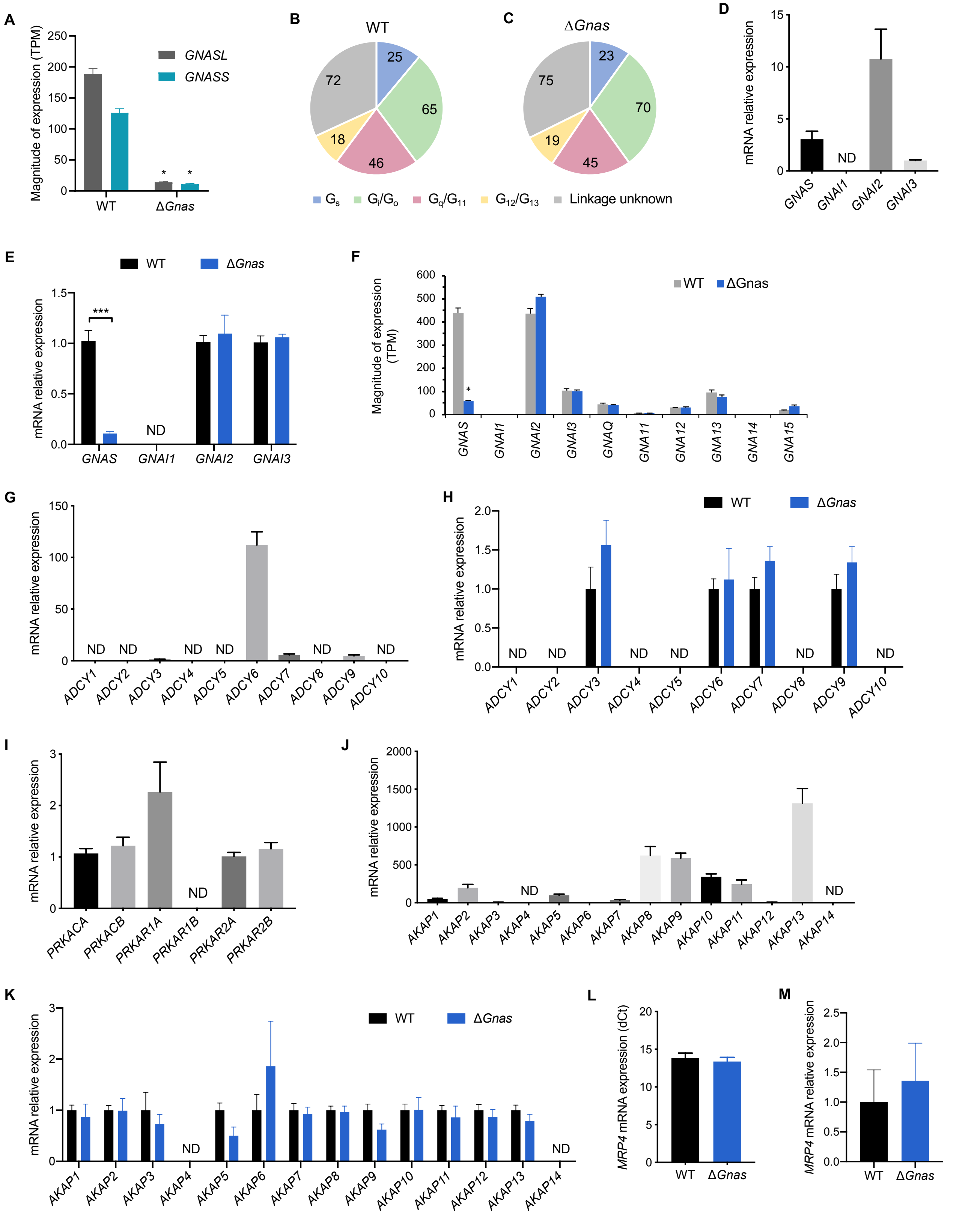
A
Magnitude of expression (TPM)
GNASL
GNASS
WT
ΔGnas
B
WT
C
ΔGnas
25
65
46
18
72
23
70
45
19
75
Gs
Gi/Go
Gq/G11
G12/G13
Linkage unknown
D
mRNA relative expression
ND
GNAS
GNAI1
GNAI2
GNAI3
E
WT
ΔGnas
***
ND
F
Magnitude of expression (TPM)
GNAQ
GNA11
GNA12
GNA13
GNA14
GNA15
G
H
ADCY1
ADCY2
ADCY3
ADCY4
ADCY5
ADCY6
ADCY7
ADCY8
ADCY9
ADCY10
I
PRKACA
PRKACB
PRKAR1A
PRKAR1B
PRKAR2A
PRKAR2B
J
K
AKAP1
AKAP2
AKAP3
AKAP4
AKAP5
AKAP6
AKAP7
AKAP8
AKAP9
AKAP10
AKAP11
AKAP12
AKAP13
AKAP14
L
MRP4 mRNA expression (dCt)
M
MRP4 mRNA relative expression

Supplementary Figure 1. Expression profiling of cAMP signaling pathway components in WT and Δ*Gnas* DCs. **(A)** Two well-characterized isoforms of *GNAS* are expressed at substantial levels in WT mice (> 1 TPM on average): GNASL (long) and GNASS (short) variants, also known as GNAS alpha-S2 and alpha-S1, respectively. Expression of both is significantly reduced (FDR<0.05) in Δ*Gnas* DCs. WT n=5, Δ*Gnas* n=4. **(B** and **C)** Ratio of GPCRs that are linked to different G protein families in **B)** WT and **C)** Δ*Gnas* DCs. **(D)** Gα mRNA expression profile in WT DCs; n=6. ND = not detected. Data are normalized to the lowest-expressing detectable gene. **(E** and **F)** Fold-change of Gα's in Δ*Gnas* DCs compared to WT DCs via **E)** independent qPCR; n=6, p<0.0001, and **F)** RNA-Seq; n=4-5, FDR<0.05. **(G)** Expression profile of adenylyl cyclase isoforms in WT DCs; n=3. **(H)** Fold-change in adenylyl cyclase isoforms in Δ*Gnas* DCs compared to WT DCs; n=6. **(I)** PKA mRNA expression profile in WT DCs; n=5. **(J)** AKAP expression profile in WT DCs; n=6. **(K)** Fold-change in mRNA expression of AKAPs in Δ*Gnas* DCs compared to WT DCs; n=6. **(L)** *MRP4* mRNA expression in WT and Δ*Gnas* DCs. dCt values are relative to the housekeeping gene 18S; n=3. **(M)** Fold-change in *MRP4* expression in Δ*Gnas* DCs compared to WT DCs; n=3.

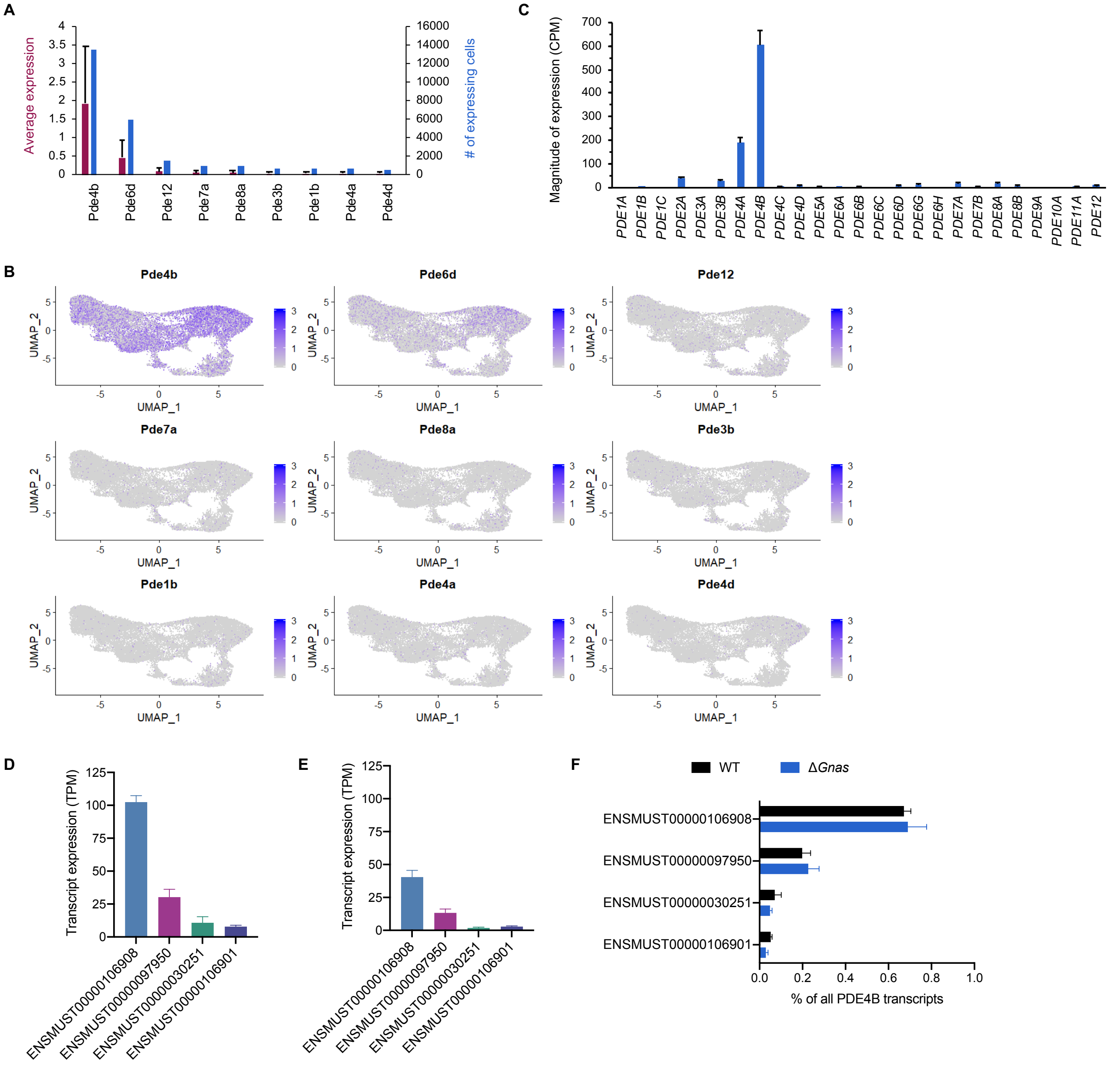

Supplementary Figure 2. *PDE4B* is the highest expressed PDE isoform in murine and human DCs. **(A** and **B)** PDE expression in single cell RNA-Seq data of murine lung DCs from (Bosteels et al. 2020) with **A)** average expression (Purple) and the number of cells expressing the gene of interest (Blue) plotted and **B)** visualized in UMAP. **(C)** PDE expression in human epidermal DC RNA-Seq data from (Bertram et al. 2019); n=4. **(D** and **E)** PDE4B transcript variant expression in TPM of **D)** WT and **E)** Δ*Gnas* DCs that were detected at mean expression levels >1 TPM in DCs from mice with each genotype. WT n=5; Δ*Gnas* n=4. **(F)** Percentage of PDE4B transcripts for each variant in WT and Δ*Gnas* DCs. WT n=5; Δ*Gnas* n=4.

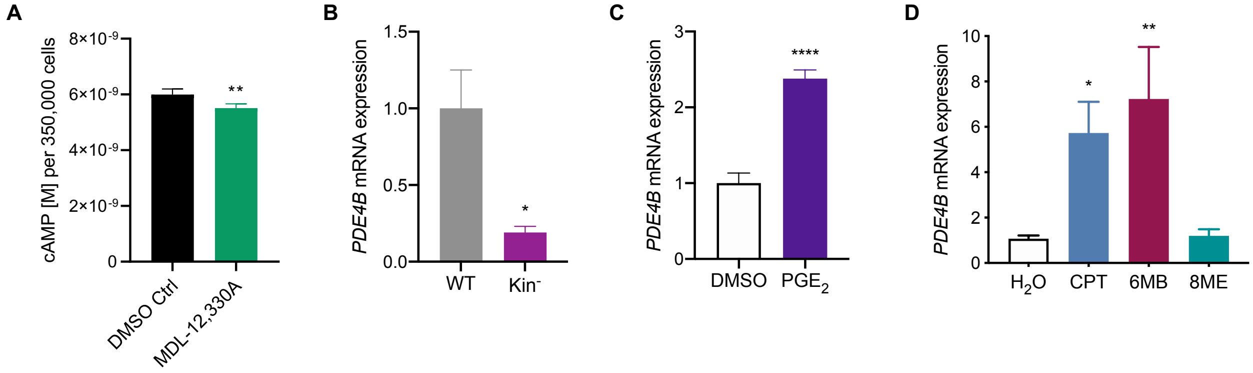

Supplementary Figure 3. *PDE4B* expression is decreased by reduced cAMP levels and lack of PKA activity and is increased in murine DC2.4 cells in response to cAMP-elevating drugs. **(A)** Decreased cAMP accumulation in WT DCs treated with the adenylyl cyclase inhibitor MDL-12,330A (10μM, 2.5 h); n=5, **p<0.01. **(B)** Basal *PDE4B* expression in murine T cell Kin⁻ S49 cells (which lack PKA activity) compared to WT S49 cells; n=3-5, *p<0.05. **(C)** *PDE4B* expression of DC2.4 cells incubated with PGE_2 (10μM, 24h); n=4-5, p<0.0001. **(D)** *PDE4B* expression of DC2.4 cells incubated with the cAMP analogs (50μM, 24 hrs) CPT (non-selective cAMP analog), 6MB (PKA-selective cAMP analog), and 8ME (Epac-selective cAMP analog); n=5-11, *p<0.05, **p<0.01.

2 Supplementary Tables

	WT 1	WT 2	WT 3	WT 4	WT 5	Avg	Std Dev
**0.2 - 1 TPM**	41	44	34	34	36	38	4
**1 - 10 TPM**	51	52	52	48	44	49	3.1
**10-50 TPM**	25	26	25	22	26	25	1.5
**>50 TPM**	15	15	14	20	14	16	2.2
**Total**	132	137	125	124	120	128	6.1

Supplementary Table 1. GPCRs expressed by WT DCs. The number of GPCRs expressed (total and stratified according to level of expression) by 5 biological replicates of WT DCs (WT 1-5).

	KO 1	KO 2	KO 3	KO 4	Avg	Std Dev
**0.2 - 1 TPM**	54	39	51	42	47	6.2
**1 - 10 TPM**	44	51	45	51	48	3.3
**10-50 TPM**	27	31	26	25	27	2.3
**>50 TPM**	16	11	13	14	14	1.8
**Total**	141	132	135	132	135	3.7

Supplementary Table 2. GPCRs expressed by Δ*Gnas* DCs. The number of GPCRs expressed (total and stratified according to level of expression) by 4 Δ*Gnas* biological replicates of Δ*Gnas* DCs (KO 1-4).

Gene	Forward Primer	Reverse Primer
18s	GTAACCCGTTGAACCCCATT	CCATCCAATCGGTAGTAGCG
ADCY1	TCCACATCACAAAGACGACC	CATCCTCTTGGCTGGTTTGA
ADCY2	CCAGTCCTACGATTGTGTCT	CACTGAACTTCGGCTTGGAAA
ADCY3	TCCATGACCAGAAAGAACGT	GGAAGCGTAGACACTCGATG
ADCY4	TTCTTCACACTCCTGGTCCT	TACGACTGGTGGTAGAGGTC
ADCY5	GGGGATTGTTACTACTGCGT	CGTCGAATTGCCACTTTCTG
ADCY6	GACCTTCCTCATACTTGGCG	TCAGGGTTCAGAGCATCTTG
ADCY7	CAGCATCGCCTTCAGTCAT	CTCGACATACACCAGCACAT
ADCY8	AGCATCACTGTCTTCGCATT	ACACCACATAGCACAGAACC
ADCY9	TTCCATTGTGATGTCCCCCT	GATCTTGGTGCGGTGTAGG
ADCY10	GGTATGGCATATCTCATCTGC	TAGTCGATCTCCCTAACACGT
AKAP1	ATGAGGTGGAGATTCGCTAC	GTGCTGTATTGCCTGTCATC
AKAP2	GCTGAAGGAGAAAGAGCCAA	TCAGTGTGGTGAGTGTGAAC
AKAP3	TTGTACTTTGCTGGTGACGA	CGTGACATTTCCCACTGCTT
AKAP4	GAAGAGCAATGCCAAGACAAT	TTCATCACGGATTCTACCAGC
AKAP5	AGAACCCAGTAATGGCATCC	TCTTCAGCAGCAGTCTTTCT
AKAP6	CAGCGAGCAGTATACCAGTG	TGTCAATCAGCCAATCCCAG
AKAP7	CACTGTGAGTCTTCCATCGT	GCTTTTTGTTCTGCGTCTCT
AKAP8	GAGAGCCCTAAACCCAAACC	AGGAATCAGCATGTCACAGG
AKAP9	GGAGTTGGAAGAGATGAGGG	TGAGTTATGATGCCGTCTCG
AKAP10	ACCAGATTGAAGTGCTGACC	TGGCTTGGAGGGAAAAGTAC
AKAP11	TGTAGACCCGCAATTAAGGAT	GACATCTCCCACTTTCCATC
AKAP11	GCAGAGAGAGAGTTGAGCAA	CTGTCTTCACCAACACTGAGA
AKAP12	CCAGTCACTCAAACAACCGA	TGTCCTCTCCATGCTGAAGA
AKAP13	GAGGATAGGGGATGTGCTTG	AGTTGACAGACTGGTTGTGC
AKAP14	ACCAGAAAAGGGGCGAAAA	CAGCATAATACACCCAGCG
EPAC1	CCTCCTGTCATCCCCTTCA	TCGGCTTCTGAGTGGTGAT
EPAC2	CTTCTGGCTCCTCCCTATGG	AAGGTCGCTGTGCTTATGTTT
GNAI1	GATTCGGCAGCGTACTATC	AGGTCATAGTCACTCAGGGC
GNAI2	CTTTGGCCGCTCACGAGAAT	GATCCACTTCTTGCGCTCAG
GNAI3	ATGGGACGGTTGAAGATTGA	CACGCCTGCTAGTTCTGAAG
GNAS	GCAGAAGGACAAGCAGGTCT	CCCTCTCCGTTAAACCCATT
MRP4	CGGACACATGGATGATTTGC	AGAACCAGGAAGACGACTGA
PDE1A	TGCTTGAGAATCACCACGTC	TTGCTGAAAATGCCCTGACA
PDE1B	GACATCAGCCACCCAACTAA	CTGGACACTCTTTTCTGCCA
PDE1C	GAGAAAAGGCAGGCGAAAAG	CGGTGTTGGAGTGATCCTTC
PDE2A	GACCGATGGAGATGATGGAC	CCACACGTTCATACAGCTCT
PDE3A	GTACGCCGAGCAGATCCT	CCTCTCTTGTGGTCCCATTC
PDE3B	GAAGAGGCACAGCAACCAAA	TCCTTCCTGATTTTTCTCCCA
PDE3B	GCTGCCTATCTTCAAACATC	ATTCTGCAAGGAAATCAAAA
PDE4A	CTCTCTGCTACCTCCACAAC	GATGAAGAGGATTCATTGGA
PDE4B	TGTCGATCATCCTGGAGTCT	AAGATGTCGCAGTGTTCCTC
PDE4C	CAACTACTCTGACCGCATCC	TAGTCAATGAATCCCACCTGG
PDE4D	CATCCTGGGGTGTCAAATCA	GTTCTCTAGGACCGAGGAGT
PDE5A	CCCTGTTCCTTGTCTGTGAG	CAACTTCTGCATTGAACCGG
PDE6A	GTACCTCAACTTTGTGAACCTGA	CTGCCTCTCTATATCCGTGA
PDE6B	TGGAGCTAGTCAAATGTGGC	TCGATAGGCTTTGCTGACAG
PDE6C	CCACAGAGGCACCAATAACT	TTCCAGGTGATGTCGCTCTA
PDE7A	ACTTACACCTTGACGATGGC	TTTCACTCCACTGCTTGCTT
PDE7A	GGAAATAGTCTAGTAAGCCTAACC	TGACTGTGGTAATCTTCTTGAAT
PDE7B	CAAGAAAAAGGTGAAACGAC	GTTGTGACCGTGGTAATCTT
PDE8A	CAAAGCGGTTTCCTCCAGAA	CTGAGCATTAAACTGTGGCG
PDE8B	TCAGTCCTTCCTCTTCTTCA	CCGGTTCTTGTCACTCTTAG
PDE9A	TTGACTACAGCAACGAGGAG	TCCAGTAAACAGTCCACCCA
PDE10A	TGAGAAAGGGATTGCTGGTC	TACCGCTGATCTTGTTCACC
PDE11A	ATCAAGTGAAGAAGTCCTGGG	CAGCTCCATGAACATCCGTA
PRKACA	CCCGAGATTATCCTGAGCAA	CACCTTCCCAGAGACGATTTT
PRKACB	CCTCCATTCTTTGCTGACCA	TTTATGTCACTCACGCCGTT
PRKAR1A	TTGTTGAAGTGGGACGACTG	CGGTCCAACTTAACGCACT
PRKAR1B	CTCGGCAGAAGTCAAACTCC	CGTCCAGGTGAGAAAAGAGC
PRKAR2A	CTGGGGAAGTGAGCATCTTG	GGGCAAGTTCTCCGAAGTAC
PRKAR2B	GTTGGAAGATGCGTCGGTAA	CTAGAGACTTGAGGAACGGC
PRKAR2B	TAACAAACCAAGAGCAGCGT	TTCATAGGTGGCGATGTTCC

Supplementary Table 3. qPCR primer sequences.
